# Health & nature: a critical review of historical perspectives to support narratives for change

**DOI:** 10.1186/s12939-025-02550-y

**Published:** 2025-10-01

**Authors:** Miquel Amengual-Moreno, Lucinda Cash-Gibson, Laila Vivas, Eliana Martínez-Herrera, Adrián Almazán, Juan M. Pericàs, Joan Benach

**Affiliations:** 1 Hospital del Mar - Agència de Salut Pública de Barcelona - Universitat Pompeu Fabra Preventive Medicine & Public Health Training Unit, Barcelona, Spain; 2 Research Group on Health Inequalities, Environment and Employment Conditions (GREDS-EMCONET), Universitat Pompeu Fabra, Barcelona, Spain; 3 JHU-UPF Public Policy Center (JHU-UPF PPC), Universitat Pompeu Fabra (UPF), UPF Barcelona School of Management (UPF-BSM), Barcelona, Spain; 4 Center for Studies On Planetary Wellbeing, University Pompeu Fabra, Barcelona, Spain; 5 UPF-Barcelona School of Management, Barcelona, Spain; 6 Ecological (Technics and Humanities) Group [(TH)ECO], Universidad Carlos III de Madrid, Madrid, Spain; 7 School of Humanities, Communication and Library Science, Universidad Carlos III de Madrid, Madrid, Spain; 8 Basque Centre for Climate Change (BC3), Scientific Campus of the University of the Basque Country, Leioa, Basque Country, Spain; 9 Epidemiology Research Group (Research Line On Epidemiology and Urban Health), National School of Public Health, Universidad de Antioquia, Medellín, Colombia; 10 Infectious Disease Department, Hospital Clínic de Barcelona, Barcelona, Spain; 11 Liver Unit, Centros de Investigación Biomédica en Red Enfermedades Hepáticas y Digestivas (CIBERehd), Vall d’Hebron University Hospital, Vall d’Hebron Institute for Research (VHIR), Barcelona, Spain; 12 Universitat Pompeu Fabra, Barcelona, Spain

**Keywords:** Concept of health, Critical review, Environment, Nature, Sustainability, Ecosocial crisis

## Abstract

**Introduction:**

The ongoing ecosocial crisis threatens the health of our planet, as ecological boundaries are overreached and social needs remain unmet. Achieving health equity and sustainable development requires re-evaluating the interconnections between nature and health, including the social narratives shaping this relationship. The ways in which we construct and adopt these narratives—consciously or not—translates into different implications for research, policy and practice. This study critically analyses the historical evolution of how the health-nature relationship is conceptualised in the scientific literature, and classifies the different eco-social values and theoretical considerations embedded within each emerging perspective. By raising awareness of the diverse perspectives used and their implications for research, policy and practice, the findings aim to provide a conceptual guide for narratives that aim to drive change towards health equity and sustainable practices.

**Methods:**

We conducted a critical review to identify the main perspectives of the health-nature relationship in the scientific literature over the past 60 years, and to categorize them based on their ecological theoretical positions, ranging from anthropocentric to non-anthropocentric. Snowballing techniques were applied to include other relevant literature.

**Results:**

Our review identified eight main perspectives on the health-nature relationship during this time period: Environmental health, Ecology of health, Holistic medicine, Political ecology of health, Eco Health, One Health, Planetary Health, and Indigenous traditions. We then classified them by their consideration of nature, and ecological positions.

**Discussion and conclusions:**

Our results found diverse and evolving perspectives on the health-nature relationships, with anthropocentric to non-anthropocentric ecological theoretical positions embedded within them. When selecting and applying perspectives to support transformation, researchers and policymakers should have a clear idea of the implicit and explicit theoretical positions embedded within them. Researchers, policy makers, and practitioners should carefully consider these findings when selecting frameworks to guide narratives of change, and interventions aiming to address the political, ecological, economic, and cultural drivers of environmental degradation, human and natural exploitation, and social and health inequalities that our planet is struggling with. Recognizing these varied perspectives presents an opportunity to embrace diverse epistemologies that can inspire positive ecosocial change and foster a more sustainable and equitable relation between human societies with nature.

**Supplementary Information:**

The online version contains supplementary material available at 10.1186/s12939-025-02550-y.

## Introduction

The ongoing ecosocial crisis threatens the health of our planet. This necessitates a fundamental epistemological shift to re-evaluate our actions linked to our understanding about the interconnected health-nature relations. Throughout history, different understandings of health (and illness) have emerged, which have been shaped both by material realities (i.e., biology, economic drivers, energy resources, infrastructures, historical events, and environmental contexts) and by societal views or imaginaries of how society does and should function (i.e. cultural constructs, religious influence, and technological imaginaries) [13, 18, 41, 44]. These different understandings have underlying values and sociopolitical assumptions [62], and entail particular forms of measurement, analysis, and interpretation of health outcomes, as well as research, policies, and actions aimed at improving health [44, 53, 62, 106].

**Fig. 1:**
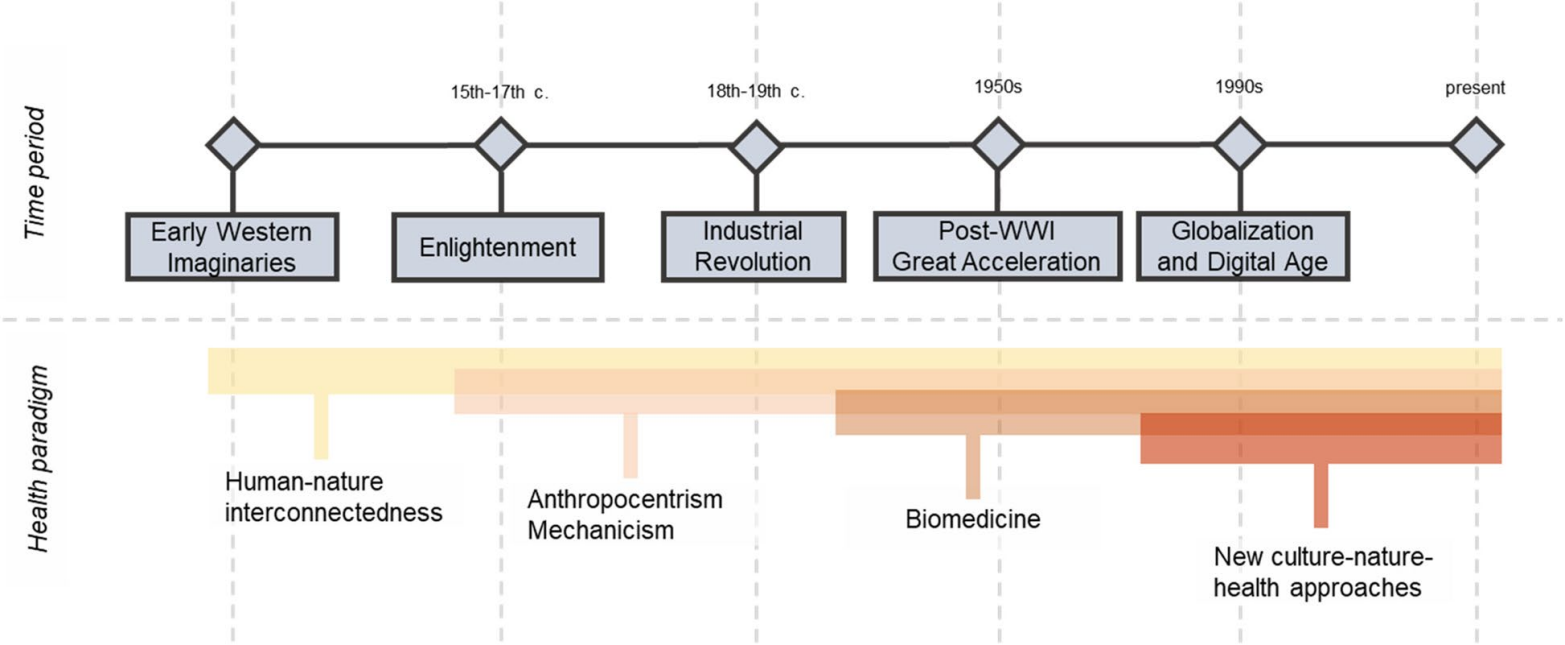
Historical Evolution of Western Health-Nature-Society Relationships

In this regard, perspectives on health and its relationship with nature have changed along history. Throughout different historical epochs, societies had attached varied meanings to the concept of nature, which in turn have influenced the way human health has been considered in relation with nature. Figure 1 presents a historical overview of the evolution of western health-nature-society relationship.

For instance, early Western perspectives often emphasised human-nature interconnectedness and a related holistic sense of health, involving environmental influence [29, 44, 52, 61]. However, during the Enlightenment period (fifteenth—seventeenth centuries), there was a significant shift in how society viewed and interacted with nature, and anthropocentric and desacralized views emerged. Nature was portrayed mechanistically and controllable by human mastery and reason [40, 65] leading to explanations of the human body based on mechanical principles [29]. The Industrial Revolution (eighteenth—nineteenth centuries) further solidified these transformations, emphasizing health interventions focused mainly on isolated elements like germs or sanitation. There were advancements in fields like epidemiology and germ theories, alongside the rise of hygienism and state interventions in response to the spread of diseases in densely populated industrialised cities [45, 77]. Such historical processes contributed to the contemporary biomedical conception of health [58, 61, 74].

During the post-World War II era, the World Health Organization (WHO) introduced a widely recognised definition of health in 1946, which states that health is not merely the absence of disease or infirmity but a state of complete physical, mental, and social well-being [116]. This definition emerged along with the establishment of the Welfare State and a new social contract influenced by working-class movements [109]. Although the WHO definition has been criticised for its practical limitations [53], it served as a starting point for expanding the understanding of health beyond mechanism, sometimes influenced by ecological perspectives, which sought to integrate concepts like ecological well-being into the definition of health [50, 61, 86].

Since the 1950 s, a boosted capitalist economy, global economic growth, population increase, and resource consumption have developed, representing a profound shift in the human-nature relationship in what has been called the Great Acceleration [104]. In this time period, Earth Systems (i.e., the geosphere, biosphere, cryosphere, hydrosphere, and atmosphere) have experienced unexpected changes from historical norms [103, 104]. Some scholars refer to this period as the Anthropocene [25], closely aligned with the Great Acceleration [103], while others suggest labelling this epoch as the Capitalocene [67] to signal the responsibility of capitalist processes.

During the later part of the twentieth century, the emergence of diseases linked to urbanization and industrial expansion highlighted the limitations of mechanical philosophy applied to non-communicable diseases, giving rise to the multicausality models to account for multiple risk factors [69, 101]. The influential Lalonde Report from Canada, which focused on quantifying exposure to environmental risks while limiting the role of nature to such risks, is a paradigm of the latter [48, 58].

While dualist approaches to health and nature are dominant in the Anthropocene [26], the impacts of nature degradation resulting from the Great Acceleration have led to the (re)emergence of imaginaries of nature based on eco-dependence, which recognise humans as a part of nature. Starting in the 1950 s, literature highlighted the consequences of rising carbon dioxide levels and fossil fuel burning on the Earth's climate [71], the harmful effects of biocides on the environment [14], and discussed “*The Limits to Growth”* [64]. The development of environmental justice movements and principles linked human health and the health of the environment to social inequity [2, 5, 32]. These influential publications and growing social movements have led to an increased recognition of how important the interdependent relationship between humans, nature, and health is, and further propelled ecological concerns into the public agenda. The Gaia theory by James Lovelock, for example, proposed the Earth as a self-regulating superorganism, and symbiogenesis by Lynn Margulis, who pointed out that complex cells evolved through the merging and integration of different species in a mutually beneficial symbiotic relationship [56, 57, 92].

Throughout history, Indigenous cultures and knowledge systems have also developed their own perspectives on the relationship between health and nature that emphasise harmony, and intertwine spirituality to create more holistic and community-based approaches to health that consider both individual and collective well-being. These perspectives represent enduring and evolving traditions of knowledge that continue to guide health and environmental practices. Indigenous scholars have called for the decolonization of health and environmental sciences, challenging extractivist paradigms and asserting holistic frameworks and narratives that prioritize interconnected well-being across generations and ecosystems [3, 21]. However, these perspectives have not often been incorporated into Western scientific perspectives of health and nature.

More recently, the United Nations selected 17 universal objectives—the sustainable development goals (SDGs)—to transform the world into a more sustainable place by 2030. The SDGs seek to respond to climate change, improve the management of natural resources, reduce inequalities, and improve health and wellbeing for all. Nonetheless, there are doubts about whether these goals can truly be achieved, due to the implicit and explicit theoretical and ideological positions embedded in these narratives, goals and targets [105].

Understanding the evolution of the conceptualisations of health and its relationship with nature, and the different implicit and explicit values and theoretical considerations embedded within these perspectives, is important, since they have different implications in terms of how to safeguard nature and health in practice. Several literature reviews exist on conceptual frameworks for health and nature, some focused on the literature since the 1990 s and 2000 s, and others with historical insights in the literature that provide a really valuable insight into the ecological evolution of the conceptualizations of health [12, 55, 58, 80]. However, none of these papers have attempted to analyse and classify the different implicit and explicit eco-social values and theoretical considerations embedded within each perspective that has emerged. This additional knowledge could be useful to raise awareness of the importance of critically analysing perspectives and narratives that have emerged on health and nature over time, and guide future research and policies on health equity and sustainability. This study aims to fill this gap by critically analysing these perspectives.

## Methods

We conducted a critical review of scientific literature that has conceptualised the relationship between health and nature since the onset of the Great Acceleration in 1950 to date, to provide a descriptive account of the historical evolution of the main perspectives on the health-nature relations [36, 107]. This time frame is important because it is the moment when all Earth trends start to drastically change, and it will be interesting to observe whether and how these changes have been reflected in the scientific literature. Furthermore, we classified the main perspectives found according to the environmental ethics thought on the health-nature relation mentioned in the documents to make their underlying theoretical-ecological position more explicit [60].

### Search strategy

We conducted a search for relevant literature in PubMed and Scopus databases. PubMed was selected since it indexes core medical journals from the 1960 s onwards, while Scopus was chosen due to its extensive journal coverage across health, life, and social sciences, as well as its comparability with bibliometric analysis [33]. We defined the search strategy to be as sensitive as possible to identify literature regarding conceptions of health and nature. The following key terms were used related to HEALTH and NATURE: ((“defin* of health”) OR (“defin* health”) OR (“concept* of health”)) AND ((“ecology”) OR (“natural”) OR (“ environment”) OR (“ecosystem*”) OR (“sustainab*”)). Snowballing techniques were also applied to include other relevant literature identified through secondary references and preliminary research references.

### Inclusion criteria:

1. All types of documents (papers, articles, reviews, books, commentaries, etc.) that provide an insight into the conceptualization of health and nature, as defined by the authors of the documents. (Note. this information was found once we screened the full texts).
2. Publications written between 1950 and to date.
3. The full text of the publication is available.

### Exclusion criteria:

(1) The conceptualization of health and its relationship with nature is not explicitly mentioned in the text. This includes empirical papers that use a certain framework but do not theorize about them (e.g. a paper using One Health to analyse a certain problem, but that does not provide new insights).

(2) Human health is not referred to in the text.

(3) Publications written before the 1950s.

### Literature selection

The search was conducted in January 2025. The screening process was conducted by three authors (MAM, LCG, LV); firstly, each publication’s title and abstract were independently reviewed, and publications that did not meet the inclusion criteria were excluded from further analysis. Secondly, the full texts of the remaining publications were obtained, and reviewed. Relevant publications that met the inclusion criteria were included in the review and were later classified. Any doubts or uncertainties over the documents found were discussed between the three researchers, and with other members of the research team until consensus was reached. Periodic (weekly) encounters were held to ensure the continuity of the discussion during this stage of the process.

### Conceptual classification

Marcos’ [60] classification was used to broadly classify the ecological ethics position within each health-related perspective according to the type of relationship established with nature, and makes the theoretical positions more explicit. This framework offers a structured lens to understand the type of ethical relationship established with nature within each health-nature perspective, making explicit the often implicit ecological ethics that inform different health approaches. This classification was not used as an inclusion or exclusion criterion, but rather as a conceptual tool to situate and interpret the theoretical foundations of the materials included in our analysis. It helped us map the underlying values attributed to nature across the selected literature and assess how these values influence different visions of health and well-being. The broad classification used is as follows:

o *Heavy (or strong) Anthropocentrism*: this perspective asserts the absolute primacy of humans over nature, neglecting any moral consideration of the relationship between humans and the natural world.
o *Light Anthropocentrism*: while still placing human interests at the forefront, this perspective recognises that nature holds value beyond its economic utility and acknowledges humans as responsible and rational administrators of nature. However, nature is primarily seen as a resource to be managed for human benefit.
o *Biocentrism*: this perspective acknowledges the moral relevance of all living beings. It considers the capacity of beings to feel or experience pain as a basis for their moral consideration. Biocentrism recognises that living beings have interests and goals, and as such, they possess intrinsic value. However, intrinsic value does not necessarily equate to having rights.
o *Ecocentrism*: extends moral consideration not only to individuals but also to entire ecosystems. Its main difference with biocentrism is that it recognises that all entities, regardless of their consciousness or agency, hold intrinsic value. This includes the ecosystems itself as a moral entity with rights. Ecocentrism emphasises the interconnectedness and interdependence of all elements within an ecosystem.

Since the purpose of this study was to conduct a critical review—focusing on the critical conceptual contributions found in the articles—we include the search information here rather as part of the results. 1061 references were retrieved from the databases, and duplications were eliminated. Following the screening of titles and abstracts, and full text review, 88 references remained. In addition, 20 additional references were drawn from snowballing techniques and specific searches were included in the review. 108 publications were then included in this critical review. In the supplementary material we provide a list of all documents included. Each source was reviewed and classified based on the main health-nature approach and the dominant ethical orientation reflected in the argumentation or framework. This required interpretive judgment; rather than applying the classification rigidly, we used it to illuminate the normative assumptions embedded in different health-nature relationships.

## Results

**Fig. 2:**
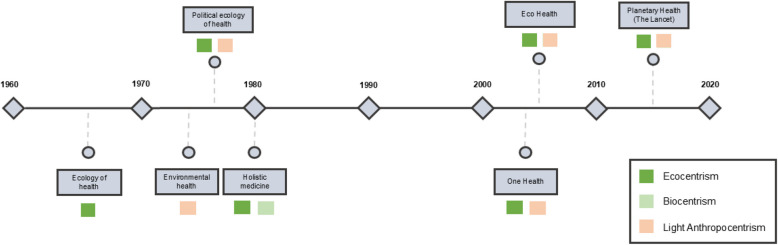
Timeline showing the emergence of the different Western scientific nature-health perspectives

Through our critical review, we identified eight main perspectives on the conceptualization of health and its relation to nature: Environmental health, Ecology of health, Political ecology of health, Holistic medicine, Eco Health, One Health, Planetary Health, and Indigenous perspectives. The historical emergence of Western scientific perspectives is represented as a timeline in Fig. 2. Time of emergence is set as the first time the perspective appears in the literature included in this critical review. Also, perspectives could have emerged prior to our critical reviews timeline (pre 1960 s). Indigenous perspectives are not represented in the Figure because it is difficult to determine exactly when they emerged, and would require further in depth analysis beyond the scope of this review.

Between the 1960 s and the 1980 s, we identified three main perspectives that emerged in the literature: Ecology of health, Political ecology of health, and Holistic medicine which acknowledge ecosystems and emphasise the importance of considering the entire biosphere in health studies. We therefore consider that they align with Marcos’ [60] non-anthropocentric position regarding the health-nature relationship, which goes from biocentrism to ecocentrism. More specifically:

**Ecology of health** perspectives seem to focus on body-environment dynamic equilibrium. Within this perspective, Health appears to be viewed as an ongoing transaction with the environment, emphasising the interconnectedness of human well-being and ecological systems [4, 28, 29, 49, 93, 94, 101, 102, 108]. These perspectives recognise society and its interrelations as an integral part of the human environment [4, 17, 27, 28, 94]. References to the value of ancestral and Indigenous knowledge can be found within these approaches [94], which remain far away from the health-nature dualism [86, 94], this is, from establishing a separation between humanity and nature. Following Marcos [60] classification, we would classify ecology of health to be most related to ecocentrism.

Within **Political ecology of health**, there are notable approaches such as the political ecology of disease, rooted in Marxist theories [61, 84, 109], which emphasise the means of production as a determinant of health [61, 75, 109] and advocate for the recognition of the role of politics and power relations, as well as overcoming the human–environment dichotomy through the analysis of social relations [75]. For some authors using these approaches, human–environment relations are characterised by a dominion of nature, as the labour required for human survival becomes central [61, 109]. Within the realm of political ecology of disease, proposals stemming from environmental justice movements adopt an integral vision of health encompassing *“…not only illness and death, but also life, nature, culture and fundamental human rights”* [84], and the capacity of communities affected by environmental injustices to democratically address and overcome these issues [84]. We therefore consider most political ecology of health perspectives to align with what Marcos [60] classifies as an ecocentric position towards nature, with some aligning with light anthropocentric positions.

**Holistic medicine** perspectives seemed to have emerged in the literature in the 1980 s as a movement against mainstream biomedical medicine and its neglect of the environment and social conditions’ effects on health. These perspectives encompass various approaches, which differ on its scientific basis, and present philosophical assumptions that diverge from mainstream scientific medicine, challenging body-mind dualism and integrating spirituality and ancestral knowledge into medical practice. The growing recognition that environmental degradation directly causes many diseases—and that conventional medicine is ineffective at preventing them—spurred Holistic medicine’s growth [11]. However, others argued that some Holistic medicine’s individualistic approaches neglect the social causes of disease and may overly emphasise individual healing strategies, potentially leading to medicalisation, while overlooking the socio-economic and political contexts surrounding health [4, 11]. Given the heterogeneity found, it was challenging to classify this perspective into just one of Marcos’ [60] classifications, however, we consider that it tends to align with non-anthropocentric positions, from biocentrism to ecocentrism.

Also in the 1970 s, the **Environmental health** perspective emerged, which aimed “*to assess the influence of various factors on the general level of illness*” [48], and became hegemonic [6]. This perspective portrays nature as a combination of environmental exposures and risks, individuals’ characteristics and the “way of living”, influencing health through multicausal interactions [61, 69], its goal is to identify and mitigate environmental risks to health through public health interventions. It primarily focuses on exposures, considering them as contributing factors rather than underlying causes of disease [11]. Environmental health has gained criticism due to its deterministic view of health [37] and how it does not consider broader ecosystems,we therefore classify it as light anthropocentric.

Since the 1990 s, Eco Health, One Health, and Planetary Health perspectives also emerged in the literature. While they share similarities, they also hold distinct perspectives on the conceptualization of health and its relationship with nature, and there is a high variety of positions within each approach. For instance, the application and interpretation of Eco Health, and One Health appear to significantly differ among researchers. While some researchers may focus primarily on zoonotic disease transmission [113] and the interconnectedness of human, animal, and environmental health, others may take a broader perspective that encompasses social determinants of health and the impacts of environmental degradation on overall well-being [38]. The theoretical positions of these three main perspectives appear to range from light anthropocentrism to ecocentrism.

**Eco Health** perspectives, and an ecosystem-based health approach -which differ from other approaches as they tend to focus on health as a property of ecosystems, not only individuals- emerged alongside the United Nations Millennium Development Goals, and from the influential document “*Ecosystems and Human Well-Being: Health Synthesis*” within the Millennium Ecosystem Assessment [24]. Eco Health is considered to foster *“…the health of humans, animals and ecosystems”,* and claim to recognize “…*the inextricable linkages between the health of all species and their environments”* [55]. Eco Health perspectives appear to value socio-political relationships in health and recognise the importance of incorporating ancestral knowledge [12, 20, 38, 55], viewing health as an attribute of ecosystems, not just individuals, promoting an ecocentric outlook [55]. Also, they appear to acknowledge the interdependence of humans, wildlife, and ecosystems, emphasising transdisciplinarity, involving humanities, social sciences, and health-related disciplines [12, 20, 55, 58], to address the complex challenges at the intersection of health and the environment [38, 55]. Some critical Eco Health approaches also consider power relations influencing ecosystems [38]. Eco Health recognises the intrinsic value of ecosystems [20], and could be classified as non-anthropocentric based on Marcos’ [60] classification. However, its understanding of them often revolves around the notion of “ecosystem services” [24], which when brought into practice, vary in their human-nature position, being some of them anthropocentric, despite the majority of examples are community-built and non-anthropocentric [20].

**One Health**, rooted in the One Medicine movement of the nineteenth century [54], has gained prominent recognition in more recent years, particularly in response to emerging zoonotic diseases. It encompasses a wide array of perspectives and approaches, ranging from a narrow focus on the convergence of human and veterinary medicine for disease control to a more holistic view that incorporates social sciences and expands its scope [26, 38]. In 2009, a One Health Commission was established, and defined One Health as *“the collaborative effort of multiple health science professions […] to attain optimal health for people, domestic animals, wildlife, plants, and our environment”* [82]. A recent definition of One Health, from the One Health High-Level Expert Panel, goes further to emphasise the importance of ecosystems. According to this definition, One Health is described as *“an integrated, unifying approach that aims to sustainably balance and optimize the health of people, animals, and ecosystems. It recognizes the health of humans, domestic and wild animals, plants, and the wider environment (including ecosystems) are closely linked and inter-dependent”* [1]. This definition signifies a broader interdisciplinary approach, similar to some Eco Health perspectives. However, unlike Eco Health, One Health often focuses on health as a property of individuals, whether human or animal, primarily involving health science professionals, and ignores individual, population and ecosystem dimensions of health [55, 96]. Thus, the concept of One Health, and the notion of health itself beyond One Health, remains unclear and its theoretical foundations vague [96]. In line with Marcos’ [60] classification, its conceptualisation of the nature-society relationship therefore ranges from anthropocentrism to biocentrism, as they draw attention to non-human health instrumentally for the benefit of human health only [6, 96], and appear to lack recognition of the significance of social relations in health and environmental degradation [26]. The first proposals on the definition of One Health ignored wildlife health, and were focused on livestock and humans, considered wildlife in terms of a threat to health because of its zoonotic potential when in contact with humans and livestock [35]. Meanwhile, other positions acknowledge sentient beings and their well-being, and in its most recent definition, also ecosystems.

While we found no precise definition of ***Planetary Health*** in the 1970s. In 2015, the Rockefeller Commission on Planetary Health defined Planetary Health as: *“the achievement of the highest attainable standard of health, well-being, and equity worldwide through judicious attention to the human systems -politic, economic, and social- that shape the future of humanity and the Earth’s natural systems that define the safe environmental limits within which humanity can flourish”* [115]. The Commission claims originality of the term [115], and many scholars have since referred to the Rockefeller Commission on Planetary Health definition. However, the term “Planetary health” was used in the literature prior to this date. Early notions of the perspective acknowledge ecosystems and higher entities, and emphasised the importance of sociopolitical relations and valued Indigenous and ancestral knowledge. Jonas Salk, a prominent American virologist and medical researcher, defended Holistic Medicine and is considered to be one of the first to discuss the idea of Planetary Health [68, 86]. Other authors have called for the establishment of a new interdisciplinary field of science known as “planetary public health” [108], stressing the inseparability of human and planetary health [86], highlighting the impact of human activities on the “planetary life support-system” [86, 95], which were aligned with ecocentric positions. Within most recent definitions and approaches of Planetary Health, we consider that anthropocentric, ecocentric and biocentric positions can be found. Several authors have also considered the Rockefeller Commission’s perspective to be anthropocentric, as it primarily focuses on human health and threats to it [55]. Some initiatives in the last years have attempted to expand Planetary Health to include Indigenous perspectives that lean towards ecocentrism. Despite this, it has been criticised for a lack of incorporation of social sciences [42] and post-colonial and decolonial views [85].

Furthermore, we also identified **Indigenous and ancestral perspectives within the documents found,** which seem to have been discussed and incorporated into Western scientific literature since the 1980 s on health and nature [19, 73, 90, 97]. Papers that include these perspectives often converge in recognising that health encompasses more than the mere absence of disease, but rather it is deeply intertwined with the well-being of the environment, the harmonious relationship between humans and nature, and the emotional connections individuals form with the natural world. Spirituality and harmony also hold a central place in their perspectives [46, 76, 81, 99, 114, 117] Some of the scientific paradigms here reviewed like ecology of health and Eco Health have recognized these views in their theories. Therefore, in line with Marcos’ [60] classification, we would classify these perspectives found as non-anthropocentric, with a tendency to align with ecocentric positions. However, our review probably only identified a few of these perspectives through western scientific literature. Any conclusion or generalisation from our results about Indigenous perspectives of health should not be done, as it would require anthropologic and ethnographic methods for each Indigenous people that are far beyond the scope of this paper.

## Discussion

**T**here is a growing global awareness of the need to overcome the challenges of the Anthropocene in order to safeguard both our health and the health of our planet. This includes an increasing recognition that we must act in a more sustainable and equitable manner, we must critically re-evaluate our current relationship with nature, and acknowledge and act on the root causes of illness and health inequalities that can be found upstream in the social, political, and ecological determinants of health [70]. Through the emergence and use of health-nature perspectives, narratives for changes can be stimulated and promoted [31]. However, each perspective carries implicit ecological values and theoretical positions that have meaningful implications for future research, policy and practice if used [79]. It is therefore important to make these values and positions explicit. This is what our critical review attempted to do. Surprisingly, our critical review found a high variety of polysemy between and within the eight main perspectives on the relationships health and nature that appear to have emerged in the scientific literature since the 1960 s, with different implicit theoretical-ecological positions ranging from light anthropocentrism to ecocentrism.

We found that numerous definitions appear to exist per perspective, and scholars lack precision when stating which perspective they use and why, which can create confusion when selecting a perspective to use, and therefore potentially hinder meaningful changes towards creating more sustainable, equitable and healthy environments [91]. Therefore it is important for researchers and policy-makers to be clear about the implicit and explicit considerations behind the terms selected and used in research, advocacy and decision making.

Marcos’ [60] classification was used to broadly classify the ecological ethics position within each health related perspective according to the type of relation established with nature, and makes the theoretical positions more explicit. Considering this classification, light as well as heavy or strong anthropocentric positions maintain the assumptions of the age Enlightenment period occurred in Europe in the 17th and the eighteenth centuries, considering human dominion over nature, positioning such realms as separate entities and consequently establishing nature-culture dualism [51].

Meanwhile, non-anthropocentric positions such as biocentrism and ecocentrism hold completely different assumptions about how society interacts with nature, as they recognise sentient beings and, in the case of ecocentrism, higher entities composed by living beings and the whole environment, at the same level of relevance as humans. When applied to health, an anthropocentric approach to an emerging disease, as One Health, may acknowledge the relevance of animal health, but only in terms of the benefits to human health [96], without discussing more profoundly the implications in which societies may interact and understand nature, or without recognizing health at the ecosystemic or planetary level [110].

We did not identify any heavy or strong anthropocentric positions within the analyzed perspectives on health-nature. However, this does not necessarily mean that dualist ideas have been overcome. Light anthropocentric positions acknowledge the environment, although often viewing it in terms of “exposures” or “risks” instead of an interconnected reality composed by intricate and complex nets of biodiversity, and still have some element of dualism in their core [6].

In the 1960 s, except for René Dubos and his vision of human-nature interconnectedness [28, 29], the visions of nature and health were mainly anthropocentric, influenced by this classic epidemiology and the Environmental Health perspectives. In the 1970 s, it appears that there was a growing recognition for an ecological approach to health, challenging previous anthropocentric perspectives, which emphasised the importance of an ecosocial approach, acknowledging the interconnectedness of humans and nature [93, 101, 102]. New perspectives such as Ecology of Health or Political ecology of health also emerged, drawing upon notions of balance, adaptation, equilibrium, and interdependence that previously existed in Western, ancestral, and Indigenous traditions [23, 112], which appeared to be more ecocentric-oriented. This turn towards ecological perspectives coincided with a broader rise in environmental awareness, both within civil society and in the international policy arena. Key developments in the early 1970 s included the establishment of the United Nations Environmental Programme in 1972 and the publication of *The Limits to Growth* by Meadows et al. that same year [7, 64]. Meanwhile, environmental justice movements, especially in the United States, began exposing the entanglements between health, environment, and socio-economic-ethnic inequalities. A significant early example was the Memphis Sanitation Strike in 1968, part of the Civil Rights Movement, which highlighted the racialized dimensions of environmental and occupational health injustices [43]. Further on, in the 1980 s, environmental justice movements expanded, particularly after the mobilizations in Warren County, North Carolina, in 1982 [87, 98]. Research on health and environment in the 1970 s and 1980 s in the Global North was thus influenced by these events and awareness.

Several scholars who have used Ecology of Health perspectives have called for a reformulation of the WHO’s definition of health, shifting away from the (bio)medical model of health [109] to broader socio-political models of health. As this acknowledges that the health systems alone are unable to diminish and prevent disease burdens [93]. These approaches criticised the dualistic approach of physicians [29] and called for new interdisciplinary approaches like planetary public health and Planetary Health [68, 108]. This emergence could be linked with ecologist and environmental movements that flourished up in the 1970 s and raised interest in this field by researchers in several disciplines [2, 5, 32].

Between the 1980 s and the late 1990 s, without new horizons of health and nature-society, no new perspectives seemed to have emerged in the literature. A possible explanation is that environmental health perspectives became paradigmatic since its emergence in the 1970 s [6], and it was not until the late 1990 s when new international consensus documents and narratives emerged (i.e. regarding the MDGs and the concept of sustainable development) that new perspectives were fostered. The geopolitical context of the 1990 s was marked by the solidification of United States hegemony, following the collapse of the Soviet Union, and the global expansion of neoliberalism, reinforcing state-corporate alliances. Additionally, in the 2000 s, security concerns became increasingly dominant, particularly after the 9/11 attacks, extending into health policy—especially in relation to infectious disease [22, 39, 47].

In this context, we found that Eco Health and One Health perspectives arose in the literature in late 1990 s and early 2000s. These perspectives might have emerged before the 1960 s, but it is out of our research scope. While they expanded the concept of health, they often display fragmented and anthropocentric perspectives on society-nature relations, often valuing ecosystems and non-human health in relation to the services they offer to humans [38, 96]. In the literature, Eco Health appears to be frequently used interchangeably with One Health primarily focusing on the management of zoonotic diseases [38]. Some authors pointed out that One Health involves various concepts, and that this vagueness could lead to contradictory aims between them [6, 96]. For other authors, One Health seems to be founded upon an apolitical conception of human, animal and environmental health, and called for expanding One Health scope to include non-positivist perspectives, and incorporate humanities and social sciences [26, 38], or wildlife health in a non-anthropocentric sense [35]. A 2022 bibliometric analysis of One Health publications show that the vast majority of them come from Europe and Central Asia, with a few of them from Asia or Africa [88]. This suggests that One Health is a heavily westernized framework.

Since the 1990 s, these new approaches fostered fruitful and integrative research in health, offering valuable contributions to addressing urgent concerns like zoonosis. However, instead of providing meaningful insights into new narratives for change of health, they often prioritised the promotion of collaborative and multidisciplinary efforts to tackle emerging health threats whilst remaining apolitical and ignoring the structural determinants and power systems that shape health outcomes. Furthermore, the interdisciplinarity of the One Health concept is in discussion, as it appears to have silos between the different disciplines researching it [59].

Over the last decade, Planetary Health perspectives appear to have acquired a great widespread influence, particularly since the 2015 Lancet-Rockefeller report on Planetary Health [115]. To our view, the report and definition on Planetary Health fails to acknowledge previous ideas related to this term, insufficiently acknowledges the the political, economic and social values and systems that shape of humanity, health and the Earth’s natural system, and politely avoids the issues of exploitation, discrimination, and extractivism embedded in these systems. Also, some authors consider that this understanding of Planetary Health primarily targets health professionals, which undervalues the role of other relevant stakeholders and disciplines that can play a key combined role in achieving Planetary Health [55]. The use of language and terminology matters, as they can act as potential catalyzers of narratives for change in health [111]. Therefore, as this perspective becomes more mainstream in research, policy, and practice, unless ecocentrism and post-colonial and decolonial views are incorporated [85], there is a risk that the root causes of the planetary “illness” will not be addressed, reducing the possibilities to create meaningful change in the Anthropocene.

Despite the overwhelming evidence of the consequences of the overshoot of the planetary boundaries that has been generated since the 1970 s [63], prominent international agreements on environmental issues, such as the United Nations Conference on Environment and Development in 1992 or the Kyoto Protocol in 1997, mainly sidelined health. Over the last decade, the international cooperation scenario has introduced health in relevant agreements such as in the Climate Conference of the Parties (COPs), the Intergovernmental Panel on Climate Change (IPCC), and the SDGs and their surrounding narratives have been promoted by multilateral organizations as a relevant approach for change. Yet SDGs are anthropocentric, as they revolve around economic growth and the notion of “progress” and development, which reinforce the dominion of nature and human groups for the benefit of capital [10, 78]. This, in turn, limits the ability for the goals to be truly achieved as planned, as we are seeing in many countries [34]. Some scholars have also criticized how the SDGs have framed the concept of health, as the absence of disease, disconnected from the broader social determinants of health [100]. The IPCC has also been criticised for being unable to incorporate social sciences in its analysis of the origins of climate change [85]. So, how can we then effectively respond to climate change, to appropriately manage our natural resources, reduce inequalities, and improve health for all?

Some scholars argue that theory is central to alternative transformations or development, as it is about (re)defining current practices and imagining something different, as well as (re)directing action to the underlying causes of non-sustainable practices or underdevelopment [83]. The direction of research and practice aiming to achieve sustainable change is highly dependent on how scholars and society select and make use of certain health-nature perspectives in their narratives—which can be critical tools for change, and in the planned actions to overcome hegemonic systems of human and natural exploitation and degradation. However, it is critical to assess the implicit and explicit theoretical assumption embedded in the perspectives used, which our critical review findings attempt to support. Therefore, researchers and policymakers should reflect on their epistemological orientation, which has implications on their decisions.

Furthermore, to address the wide range of issues arising from the Great Acceleration, and to become more sustainable and equitable, it will be important to select perspectives that go beyond anthropocentrism, and the biomedical model of health [6], to recognise the interconnectedness of ecological, social, and health relations that allows us to coexist harmoniously with our wider environment. These types of perspectives must be considered to develop systemic policies to tackle exacerbating health inequalities, increasing disease prevalence, and environmental degradation [8, 9, 15, 16, 72], as well as policy impact assessments [101]. This is especially relevant in the post-COVID-19 context, where the need for a different approach to health has been made more explicit [89]. The reintegration of the notions of health and nature will require a change of the hegemonic notions of society and development, towards an ecological perspective [10], that includes health as a property of the land and greater entities, in the terms posed by Aldo Leopold [66]. Moreover, non-anthropocentric health narratives require a more horizontal dialogue between actors—including social movements and Indigenous groups—nuancing and eventually overcoming the elitization and expertation of health management. The health-nature dualism is embedded in hierarchical structures and world governance approaches that overlook, or rather instrumentalize, the daily, smaller scale, and alternative perspectives and action that are proposed and carried out from other places.

In terms of study strengths and limitations, this critical review focused on indexed scientific literature that include a conceptualization of health in relation to nature (as defined by the authors themselves) over the past several decades. The review provides a broad historical overview of the emerging main perspectives in this literature since the Great Acceleration, and classifies them according to their embedded theoretical-ecological position to highlight the implicit ecological values, which have meaningful implications for future research, policy and practice [79]. These findings were intertwined with knowledge from grey literature to offer valuable new and complementary insights for researchers, practitioners and the public interested in health, and its relationship with nature. A more detailed analysis of when and how often each perspective has been used or cited in both the scientific and non-scientific literature would require further in-depth qualitative analysis, beyond the scope of this paper. Furthermore, our analysis is mainly from Western sources, despite some literature considered Indigenous perspectives, therefore future analysis should explore these other bodies of knowledge further and see how they have been used in scientific and political discussions on health.

### Final remarks

The historical development of the last centuries have reinforced human dominion over ecological systems, and human and natural exploitation and degradation, whilst claiming to work towards health for all. Our critical review found a high variety of polysemy between and within the different perspectives on health and nature that have emerged over the last several decades, with different implicit theoretical-ecological positions ranging from light anthropocentrism to ecocentrism. This is important to be aware of since the direction of research and practice aiming to achieve sustainable change is highly dependent on how scholars and society select and make use of certain health-nature perspectives in their narratives and actions. These narratives can be critical tools for change to overcome hegemonic systems of human and natural exploitation and degradation. To create a more sustainable and equitable relationship between human societies with nature, an ecological transformation of the notion of health is certainly needed. One that reintegrates social and ecological systems, and explicitly acknowledges the interconnected global political, ecological, economic, and cultural drivers of environmental degradation, human and natural exploitation, and social and health inequalities that our planet is struggling with.

## Supplementary Information

Supplementary Material 1

## Data Availability

Data is provided in the supplementary materials.
