# Supplementary material for: Health & nature: a critical review of historical perspectives to support narratives for change
Source: Int J Equity Health. 2025 Oct 1;24:247. doi: 10.1186/s12939-025-02550-y (PMC12486497; doi:10.1186/s12939-025-02550-y)
Supplement: Supplementary file 1 — Supplementary Material 1 [file 12939_2025_2550_MOESM1_ESM.docx]

| **Authors** | **Titles** | **Year** | **Source title** | **Source** | **Document type^a^** |
| --- | --- | --- | --- | --- | --- |
| Dubos, R. | Man and his environment: biomedical knowledge and social action | 1966 | Scientific Publication of the Pan American Health Organization | Snowballing | P |
| Dubos, R. | Man, medicine, and environment | 1968 | Penguin Books | Snowballing | B |
| Stallones, RA. | El ambiente, la ecología y la epidemiología | 1971 | Publicación científica de la Organización Panamericana de la Salud | Snowballing | B |
| Sargent, F. | Man - environment problems for public health | 1972 | American Journal of Public Health | Snowballing | P |
| Lalonde, M. | A new perspective in the health of Canadians | 1974 | Government of Canada | Snowballing | B |
| Tsaregorodtsev, G. I. | Dialectics of interaction of economic and humanistic approaches | 1974 | Soviet Studies in Philosophy | Snowballing | P |
| Schlenger WE. | A new framework for health | 1976 | Inquiry | Pubmed | P |
| Turshen M. | The Political Ecology of Disease | 1977 | Review of Radical Political Economics | Scopus | P |
| Menchaca F.J. | Toward a renewed definition of health; [HACIA UNA DEFINICION ACTUALIZADA DE SALUD] | 1978 | Anales Espanoles de Pediatria | Scopus | P |
| San Martín, H. | Ecología humana y salud | 1979 | La Prensa Médica Mexicana | Snowballing | B |
| Dubos, R.. | Mirage of health: utopias, progress, and biological change | 1979 | Harper Torchbooks | Snowballing | B |
| Lidler E. | Definitions of health and illness and medical sociology | 1979 | Social Science and Medicine. Part A Medical Psychology and Medical | Scopus | P |
| Navarro, V. | Work, ideology, and science: the case of medicine | 1980 | International Journal of Health Services | Snowballing | P |
| Berliner, HS; Salmon JW | The holistic alternative to scientific medicine: History and analysis | 1980 | International Journal of Health Services | Snowballing | P |
| San Martín, H. | La crisis mundial de la salud | 1981 | Editorial Karpos | Snowballing | B |
| Lee, PR; Franks, PE | Health and disease in the community | 1981 | Möbius: A Journal for Continuing Education Professionals in Health Sciences | Snowballing | P |
| Martínez Navarro, JF | Assaig sobre salut comunitària | 1984 | Monografies de l’Acadèmia de Ciències Mèdiques de Catalunya i de Balears | Snowballing | B |
| Alonzo A.A. | Health as situational adaption: A social psychological perspective | 1985 | Social Science and Medicine | Scopus | P |
| Draper P. | Nancy Milio's work and its importance for the development of health promotion | 1986 | Health Promotion International | Scopus | P |
| Hancock T. | Lalonde and beyond: Looking back at "a new perspective on the health of Canadians" | 1986 | Health Promotion International | Scopus | P |
| Last, JM | Human Ecology and Public Health | 1987 | Prentice Hall International | Snowballing | B |
| Smirnov I. | Human health: From theory to practice | 1989 | Journal of Medicine and Philosophy (United Kingdom) | Scopus | P |
| Castellanos P.L. | On the concept of health and disease. Description and explanation of the health situation. | 1990 | Epidemiological Bulletin | Scopus | P |
| Pörn I. | Health and adaptedness | 1993 | Theoretical Medicine | Scopus | P |
| Ness P. | Understandings of health: How individual perceptions of health affect health promotion needs in organizations | 1997 | AAOHN Journal | Scopus | P |
| Waltner-Toews D.; Wall E. | Emergent perplexity: In search of post-normal questions for community and agroecosystem health | 1997 | Social Science and Medicine | Scopus | P |
| Caponi S. | Georges Canguilhem and the epistemological status of the concept of health; [Georges Canguilhem y el estatuto epistemológico del concepto de salud.] | 1997 | História, ciências, saúde--Manguinhos | Scopus | P |
| Wheatley M.A. | Social and cultural impacts of environmental change on aboriginal Peoples in Canada. | 1998 | International journal of circumpolar health | Scopus | P |
| Kovács J. | The concept of health and disease. | 1998 | Medicine, health care, and philosophy | Scopus | P |
| Piko B. | Teaching the mental and social aspects of medicine in eastern Europe: Role of the WHO definition of health | 1999 | Administration and Policy in Mental Health | Scopus | P |
| Larson J.S. | The conceptualization of health | 1999 | Medical Care Research and Review | Scopus | P |
| Conner RF, Tanjasiri SP. | Communities defining environmental health: examples from the Colorado (U.S.A.) Healthy Communities Initiative | 2000 | Reviews on Environmental Health | Pubmed | P |
| Rapport D.J. | The health of ecology and the ecology of health | 2002 | Human and Ecological Risk Assessment | Scopus | P |
| Vega-Franco L. | Ideas, beliefs and perceptions about health. A historical account; [Ideas, creencias y percepciones acerca de la salud. Reseña histórica] | 2002 | Salud Publica de Mexico | Scopus | P |
| Dierickx K. | Is nature neutral?: The concept of health | 2003 | Is Nature Ever Evil?: Religion, Science and Value | Scopus | P |
| Cecchi G.; Mancini L. | Ecosystem health and human health; [Salute degli ecosistemi e salute umana] | 2005 | Annali dell'Istituto Superiore di Sanita | Scopus | P |
| Corvalan C, Hales S, McMichael A, et al. | Ecosystems and Human Well-being: Health Synthesis | 2005 | World Health Organization | Snowballing | B |
| Mitman G. | In search of health: Landscape and disease in American environmental history | 2005 | Environmental History | Scopus | P |
| Björklund A.; Svenson T.; Read S. | Holistic and biomedical concepts of health: A study of health notions among Swedish occupational therapists and a suggestion for developing an instrument for comparative studies | 2006 | Scandinavian Journal of Occupational Therapy | Scopus | P |
| Labun E.R.; Emblen J. | Health as Balance for the Sto: Lo Coast Salish | 2007 | Journal of Transcultural Nursing | Scopus | P |
| Nettleton C.; Stephens C., et al. | Utz wachil: Findings from an international study of indigenous perspectives on health and environment | 2007 | EcoHealth | Scopus | P |
| Schramme T. | A qualified defence of a naturalist theory of health | 2007 | Medicine, Health Care and Philosophy | Scopus | P |
| Omonzejele P.F. | African Concepts of Health, Disease, and Treatment: An Ethical Inquiry | 2008 | Explore: The Journal of Science and Healing | Scopus | P |
| Yurkovich E.E.; Lattergrass I. | Defining health and unhealthiness: Perceptions held by Native American Indians with persistent mental illness | 2008 | Mental Health, Religion and Culture | Scopus | P |
| Boddington P.; Räisänen U. | Theoretical and practical issues in the definition of health: Insights from aboriginal Australia | 2009 | Journal of Medicine and Philosophy | Scopus | P |
| Dustin D.L.; Bricker K.S.; Schwab K.A. | People and nature: Toward an ecological model of health promotion | 2010 | Leisure Sciences | Scopus | P |
| Cicolella A. | [Health and environment: the 2nd public health revolution.] | 2010 | Sante Publique | Pubmed | P |
| Cicolella A. | Health and environment: The 2nd public health revolution; [Santé et Environnement: La 2e révolution de santé publique] | 2010 | Sante Publique | Scopus | P |
| Hamilton R.P. | The concept of health: Beyond normativism and naturalism | 2010 | Journal of Evaluation in Clinical Practice | Scopus | P |
| Ríos-Osorio L.A.; Salas-Zapata W.A.; Ortiz-Lobato M. | Concepts associated with health from the perspective of sustainable development; [Conceptos asociados a la salud desde la perspectiva del desarrollo sostenible] | 2012 | Saude e Sociedade | Scopus | P |
| Bullington J. | Health and Illness and Holistic Health | 2013 | Briefs in Philosophy | Scopus | P |
| Bircher J.; Kuruvilla S. | Defining health by addressing individual, social, and environmental determinants: New opportunities for health care and public health | 2014 | Journal of Public Health Policy | Scopus | P |
| Hopkirk J.; Wilson L.H. | A call to wellness - Whitiwhitia i te ora: exploring Māori and occupational therapy perspectives on health | 2014 | Occupational therapy international | Scopus | P |
| Evans B.R.; Leighton F.A. | A history of One Health | 2014 | OIE Revue Scientifique et Technique | Scopus | P |
| Green D.; Minchin L. | Living on climate-changed country: Indigenous health, well-being and climate change in remote Australian communities | 2014 | EcoHealth | Scopus | P |
| Stephen C.; Karesh W.B. | Introduction: Is one health delivering results?; [Introduction Le concept «Une seule santé» donne-t-il des résultats?] | 2014 | OIE Revue Scientifique et Technique | Scopus | P |
| Stephen C. | Toward a modernized definition of wildlife health | 2014 | Journal of Wildlife Diseases | Scopus | P |
| Lerner H, Berzell M. | Reference values and the problem of health as normality: a veterinary attempt in the light of a one health approach | 2014 | Infect Ecol Epidemiol | Pubmed | P |
| Rodrigues J.M.; Peixoto Júnior C.A. | Reflections on affirmative concepts of health and disease in the theories of Georges Canguilhem and Donald Winnicott; [Reflexões sobre conceitos afirmativos de saúde e doença nas teorias de Georges Canguilhem e Donald Winnicott] | 2014 | Physis | Scopus | P |
| Forrest C.B. | A living systems perspective on health | 2014 | Medical Hypotheses | Scopus | P |
| Ibell C.; Sheridan S.A.; Hill P.S.; Tasserei J.; Maleb M.-F.; Rory J.-J. | The individual, the government and the global community: Sharing responsibility for health post-2015 in Vanuatu, a small island developing state | 2015 | International Journal for Equity in Health | Scopus | P |
| Lerner H, Berg C. | The concept of health in One Health and some practical implications for research and education: what is One Health? | 2015 | Infect Ecol Epidemiol | Pubmed | P |
| Whitmee S, Haines A, Beyrer C, et al. | Safeguarding human health in the Anthropocene epoch: report of The Rockefeller Foundation-Lancet Commission on planetary health | 2015 | Lancet | Snowballing | P |
| Asakura T, Mallee H, et al. | The ecosystem approach to health is a promising strategy in international development: lessons from Japan and Laos | 2015 | Global Health | Pubmed | P |
| Benatar S. | Politics, power, poverty and global health: Systems and frames | 2016 | International Journal of Health Policy and Management | Scopus | P |
| Tengland P.-A. | Venkatapuram's Capability theory of Health: A Critical Discussion | 2016 | Bioethics | Scopus | P |
| Sholl J. | Contextualizing Medical Norms: Georges Canguilhem’s Surnaturalism | 2016 | History, Philosophy and Theory of the Life Sciences | Scopus | P |
| Richardson H.S. | Capabilities and the Definition of Health: Comments on Venkatapuram | 2016 | Bioethics | Scopus | P |
| Manlove, K. R., Walker, J. G., Craft, M. E., Huyvaert, K. P., Joseph, M. B., Miller, R. S., et al. | “One Health” or Three? Publication Silos Among the One Health Disciplines | 2016 | PLoS Biology | Snowballing | P |
| Pons-Vigués M.; Berenguera A., et al. | Health-care users, key community informants and primary health care workers' views on health, health promotion, health assets and deficits: Qualitative study in seven Spanish regions | 2017 | International Journal for Equity in Health | Scopus | P |
| Porto M.F.; Ferreira D.R.; Finamore R. | Health as dignity: Political ecology, epistemology and challenges to environmental justice movements | 2017 | Journal of Political Ecology | Scopus | P |
| Mallee H. | The evolution of health as an ecological concept | 2017 | Current Opinion in Environmental Sustainability | Scopus | P |
| Charlier P.; Coppens Y., et al. | A new definition of health? An open letter of autochthonous peoples and medical anthropologists to the WHO | 2017 | European Journal of Internal Medicine | Scopus | P |
| Lerner H.; Berg C. | A comparison of three holistic approaches to health: One health, ecohealth, and planetary health | 2017 | Frontiers in Veterinary Science | Scopus | P |
| Warren M. | Defining Health in the Era of Value-Based Care: The Six Cs of Health and Healthcare | 2017 | Cureus | Pubmed | P |
| SDG 2016 Collaborators | Measuring progress and projecting attainment on the basis of past trends of the health-related Sustainable Development Goals in 188 countries: An analysis from the Global Burden of Disease Study 2016 | 2017 | The Lancet | Scopus | P |
| Singer M.; Bulled N.; Ostrach B.; Mendenhall E. | Syndemics and the biosocial conception of health | 2017 | The Lancet | Scopus | P |
| Conti A.A. | Historical evolution of the concept of health in Western medicine | 2018 | Acta Biomedica | Scopus | P |
| Nordenfelt L. | Functions and Health: Towards a Praxis-Oriented Concept of Health | 2018 | Biological Theory | Scopus | P |
| Huth M.; Weich K.; Grimm H. | Veterinarians between the Frontlines?! The Concept of One Health and Three Frames of Health in Veterinary Medicine | 2019 | Food Ethics | Scopus | P |
| Wang J.; Yu R.-X.; Wang Q. | New Concept of Health with Perspective of Chinese Medicine | 2019 | Chinese Journal of Integrative Medicine | Scopus | P |
| Lerner H. | A critical analysis of definitions of health as balance in a One Health perspective | 2019 | Medicine, Health Care and Philosophy | Scopus | P |
| Prescott S.L.; Logan A.C. | Planetary Health: From the Wellspring of Holistic Medicine to Personal and Public Health Imperative | 2019 | Explore | Scopus | P |
| Harrison, S; Kivuti-Bitok, L., et al. | EcoHealth and One Health: A theory-focused review in response to calls for convergence | 2019 | Environment International | Snowballing | P |
| Spencer G.; Hope Corbin J.; Miedema E. | Sustainable development goals for health promotion: A critical frame analysis | 2019 | Health Promotion International | Scopus | P |
| Davis, A. and Sharp, J. | Rethinking One Health: Emergent Human, Animal, and Environmental Assemblages | 2020 | Social Science & Medicine | Snowballing | P |
| Mude W.W.; Fisher C.M., et al. | South Sudanese perceptions of health and illness in South Australia | 2020 | International Journal of Migration, Health and Social Care | Scopus | P |
| Pichasaca R.A. | Conception of health-disease from the cañari worldview; [Concepção da saúde-doença desde a visão de mundo cañari]; [Concepción de la salud-enfermedad desde la cosmovisión cañari] | 2020 | Revista Ciencias de la Salud | Scopus | P |
| Spiegel S.J.; Thomas S., et l. | Visual storytelling, intergenerational environmental justice and indigenous sovereignty: Exploring images and stories amid a contested oil pipeline project | 2020 | International Journal of Environmental Research and Public Health | Scopus | P |
| Guimarães M.B.; Nunes J.A., et al. | Integrative and complementary practices in the health field: Towards a decolonization of knowledge and practices; [As práticas integrativas e complementares no campo da saúde: Para uma descolonização dos saberes e práticas] | 2020 | Saude e Sociedade | Scopus | P |
| Bautista-Valarezo E.; Duque V,. et al. | Towards an indigenous definition of health: An explorative study to understand the indigenous Ecuadorian people's health and illness concepts | 2020 | International Journal for Equity in Health | Scopus | P |
| Assmuth T.; Chen X.; Degeling C.; Haahtela T.; Irvine K.N.; Keune H.; Kock R.; Rantala S.; Rüegg S.; Vikström S. | Integrative concepts and practices of health in transdisciplinary social ecology | 2020 | Socio-Ecological Practice Research | Scopus | P |
| Elton S. | Relational health: Theorizing plants as health-supporting actors | 2021 | Social Science and Medicine | Scopus | P |
| Bussalleu A.; King N., et al. | Nuya kankantawa (we are feeling healthy): Understandings of health and wellbeing among Shawi of the Peruvian Amazon | 2021 | Social Science and Medicine | Scopus | P |
| Kalra S.; Arora V.; Verma M.; Kumar V. | A novel definition of health: Crafting a contemporary classic | 2021 | Journal of the Pakistan Medical Association | Scopus | P |
| Burgos A.V.; Salcedo A.S.; Leiva G.M. | University Decolonization as a Source of a Paradigmatic Change in Health: a Necessary Reflection; [La descolonización universitaria como fuente de un cambio paradigmático en salud: una reflexión necesaria] | 2021 | Revista de Filosofia (Venezuela) | Scopus | P |
| Coope J. | On the need for an ecologically dimensioned medical humanities | 2021 | Medical Humanities | Scopus | P |
| Reweti A.; Ware F.; Moriarty H. | A tangata whenua (people of the land) approach to conceptualising Māori health and wellbeing | 2022 | Global Health Promotion | Scopus | P |
| Smith S.-J.; Penados F.; Gahman L. | Desire over damage: Epistemological shifts and anticolonial praxis from an indigenous-led community health project | 2022 | Sociology of Health and Illness | Scopus | P |
| Heuckmann B.; Zeyer A. | Science\|Environment\|Health, One Health, Planetary Health, Sustainability, and Education for Sustainable Development: How Do They Connect in Health Teaching? | 2022 | Sustainability (Switzerland) | Scopus | P |
| One Health High-Level Expert Panel, et al. | One Health: A new definition for a sustainable and healthy future | 2022 | PLoS Pathogens | Snowballing | P |
| Qiang N.; Gu S.-Y.; Wang X.-Y.; Zhang X.-X.; Xia S.; Zheng J.-X.; Gong W.-F.; Bergquist R.; Ran J.-J.; Han L.-F. | A One Health information database based on standard bibliometric analysis | 2022 | Science in One Health | Scopus | P |
| Fernández C.M. | Health from ecological justice: From the regeneration of wild nature to the prevention of future pandemics; [La salud desde la justicia ecológica: de la regeneración de la naturaleza salvaje a la prevención de futuras pandemias] | 2022 | Revista Espanola de Salud Publica | Scopus | P |
| Selter F.; Salloch S. | Whose health and which health? Two theoretical flaws in the One Health paradigm | 2023 | Bioethics | Scopus | P |
| Geltner G.; Coomans J. | The healthscaping approach: Toward a global history of early public health | 2023 | Historical Methods | Scopus | P |
| Khanal S.; Shrestha R.; Boeckmann M. | Examining health equity in Nepal’s climate change and health policies through the lens of environmental justice: insights from a content analysis | 2024 | Global Health Action | Scopus | P |
| Prah E.; Bland S. | Climate Change and Healthscapes: Toward a Postcolonial Critique for Planetary Well-being | 2024 | South African Review of Sociology | Scopus | P |
| Goulet C.; de Garine-Wichatitsky M.; Chardonnet P.; de Klerk L.-M.; Kock R.; Muset S.; Suu-Ire R.; Caron A. | An operational framework for wildlife health in the One Health approach | 2024 | One Health | Scopus | P |

^a^B=book; P=paper
